# Clustering of disease trajectories with explainable machine learning: A case study on postoperative delirium phenotypes

**DOI:** 10.1371/journal.pdig.0001267

**Published:** 2026-03-23

**Authors:** Xiaochen Zheng, Ahmed Allam, Manuel Schürch, Xingyu Chen, Maria Angeliki Komninou, Reto Schüpbach, Jan Bartussek, Michael Krauthammer

**Affiliations:** 1 University of Zürich, Zürich, Switzerland; 2 ETH Zürich, Zürich, Switzerland; 3 University Hospital Zürich, Zürich, Switzerland; Dalhousie University, CANADA

## Abstract

The identification of phenotypes within complex diseases is a fundamental component of personalized medicine, which aims to adapt healthcare to individual patient characteristics. Postoperative delirium (POD) is a complex neuropsychiatric condition with significant heterogeneity in its clinical manifestations and underlying pathophysiology. We hypothesize that POD comprises several distinct phenotypes, which cannot be directly observed in clinical practice. Identifying these phenotypes could enhance our understanding of POD pathogenesis and facilitate the development of targeted prevention and treatment strategies. In this paper, we propose an approach that combines supervised machine learning for personalized POD risk prediction with unsupervised clustering technique to uncover potential POD phenotypes. We first demonstrate our approach using synthetic data, where we simulate patient cohorts with predefined phenotypes based on distinct sets of informative features. We aim to mimic any clinical disease with our synthetic data generation method. By training a predictive model and computing SHapley Additive exPlanations (SHAP), we show that clustering patients in the SHAP feature scoring space successfully recovers the true underlying phenotypes, outperforming clustering in the raw feature space. We then present a case study using real-world data from a cohort of elderly POD patients. We train machine learning models on heterogeneous electronic health record data covering the preoperative, intraoperative and postoperative stages to predict personalized POD risk. Subsequent clustering of patients based on their SHAP feature scores reveals distinct subgroups with differing clinical characteristics and risk profiles, potentially representing POD phenotypes. These results showcase the utility of our approach in uncovering clinically relevant subtypes of complex disorders like POD, paving the way for more precise and personalized treatment strategies.

## Author summary

Our research addresses how we can identify different subtypes within complex medical conditions using advanced data analysis techniques. While traditional medical approaches often treat conditions like postoperative delirium as uniform entities, we demonstrate that they actually comprise distinct subtypes with unique underlying characteristics. We’ve developed a novel two-step approach that first predicts a patient’s risk using machine learning algorithms, then identifies the specific factors driving that risk for each individual. By grouping patients based on these personalized risk factors rather than their raw medical data, we can uncover meaningful subtypes that weren’t previously apparent. Testing this approach with both synthetic and real-world medical data proved remarkably effective. The method successfully identified distinct patient subgroups with different clinical profiles, potentially representing different forms of postoperative delirium with unique underlying mechanisms. These findings have important implications for patient care. Understanding these subtypes could help clinicians develop more targeted prevention strategies and treatments tailored to a patient’s specific condition variant rather than using a one-size-fits-all approach. This work represents a significant step toward more personalized medicine that recognizes the inherent diversity within complex medical conditions, ultimately improving outcomes through more precise interventions.

## 1 Introduction

The identification of phenotypes in complex diseases is essential for precision medicine, which seeks to personalize healthcare based on the specific traits of individual patients. This process is not only a foundation of patient-centered treatment but also paves the way for more refined, tailored therapeutic interventions. By focusing on these unique characteristics, precision medicine enhances the efficacy of healthcare delivery, making it more responsive to the diverse needs of patients. Nowadays, the recognition of distinct clinical and biological phenotypes is enhancing our understanding of how clinical manifestations correlate with underlying pathways and variability among patients [1,2]. This has been shown to facilitate the development of personalized treatment, improve diagnostic precision, and optimize patient care outcomes [3,4].

An example of such complex diseases is delirium, a serious neuropsychiatric postoperative complication that occurs in up to 46% of the general surgical population [5]. Symptoms of postoperative delirium include a rapid onset of confusion, attention deficits, disorganized thinking, and fluctuating levels of consciousness, alongside memory issues, mood swings, behavioral changes, and sleep disturbances, highlighting the need for prompt recognition and effective treatment [6]. If left untreated, it significantly raises distress, mortality rates, and the risk of long-term cognitive decline [7–10]. Therefore, efficient treatment or prevention holds a key to improving clinical management through early detection and the development of effective treatment strategies [11], which in turn can reduce healthcare costs associated with prolonged hospital stays.

Being a complex syndrome, delirium presents challenges in understanding its underlying mechanisms. Its common occurrence in the ICU and postoperative environments does not translate to a clear understanding of its pathophysiology [12]. The prevailing hypothesis suggests that delirium occurs due to disturbances in neurotransmitter balance, influenced by certain illnesses, neuroinflammatory responses, or medical treatments making its treatment and prevention complicated. Subsequently, understanding its neurobiological mechanism could provide crucial insights into brain function under stress and illness, which might shed light on other neuropsychiatric and neurological disorders [13,14].

Personalized delirium risk prediction using machine learning (ML) algorithms, trained on comprehensive perioperative patient trajectory data, has a great potential for the development of early and targeted prevention/treatment strategies. Supervised ML methods in healthcare have shown to successfully predict personalized risks for individual patient [15]. Having accurate prediction of the likelihood of delirium before and after surgery enables healthcare providers to implement early interventions tailored to particular patient needs [11]. Nonetheless, within a heterogeneous patients group, individuals may present identical risk levels but differ in their disease trajectory and development of delirium, which require different interventions [16]. This highlights a critical gap in translating ML risk prediction approaches into clinical routine and personalized intervention strategies.

Our main hypothesis is that postoperative delirium (POD) has several phenotypes that can be identified through data-driven approaches. To address this hypothesis, we propose a two-step approach. First, we develop a synthetic case study to demonstrate the feasibility of our method in identifying phenotypes within a controlled environment. Second, we apply our approach to a real-world case study of perioperative delirium to uncover potential phenotypes and gain insights into the underlying factors contributing to the development of delirium.

Our proposed approach involves three stages: (a) training perioperative prediction models (classifier) for delirium, (b) followed by the application of explainability techniques, such as SHapley Additive exPlanations (SHAP) [17–19], to assess the importance of various features. Then (c) clustering patients using the computed SHAP value explanations, to discover new phenotypes (i.e., subtypes of delirium patients) characterized by distinct clinical features for influencing this condition. Conventionally, researchers have used unsupervised clustering to discover subgroups or phenotypes before applying any classification. This cluster-first strategy has been employed to refine disease categorizations. Several recent studies have proposed methods for clustering patient phenotypes using time-series data, such as revealing new phenotypes of autoimmune diseases (Sjögren’s syndrome) with unsupervised clustering [20], representing patient trajectories in the latent features space [21], discovering predictive temporal patterns [22], learning patient representations through contrastive learning [23], optimizing clustering performance with deep learning [24], simultaneously performing clustering and classification for risk prediction [25], and leveraging semi-supervised latent temporal processes with generative modeling [26]. While such data-driven groupings can yield valuable insights, a drawback is that they are agnostic to specific outcomes or labels. The resulting clusters may not correspond to clinically relevant categories and typically require additional interpretation or labeling by experts [27]. In contrast, our proposed approach integrates prior knowledge of class labels into the clustering process. By clustering in the SHAP-derived feature space, we ensure that the groups formed are directly linked to how the prediction is made, highlighting different profiles of feature importance among the instances. This strategy capitalizes on the strength of both methods: the predictive accuracy of supervised learning and the pattern-discovery of clustering. Notably, related work has hinted at the benefits of such integration – for example, clustering on SHAP-transformed data has been shown to better separate meaningful classes or outcomes compared to clustering on raw features [28]. In summary, our approach differs from and advances beyond the traditional cluster-first pipeline by using the target-aware insights from a classifier to drive the clustering, thereby yielding more interpretable and outcome-relevant clusters. This supervised first, then cluster methodology provides a refined lens to identify subgroups that matter for the prediction task, which can ultimately enhance understanding and decision-making in the clinical context [29].

Our contributions in this paper are as follows:

**Comprehensive ML Prediction:** We propose a robust ML approach for predicting the personalized risk of postoperative delirium leveraging the heterogeneous electronic health record (EHR) data. We provide risk estimates that include the pre, intra and postoperative stages that can be used for the early detection of POD.

**Personalized Explanations:** The machine learning prediction model can be used to provide personalized explanations (SHAP values for each feature) for the development of postoperative delirium for the different stages of each patient’s journey. This approach can shed light on the individual feature contributions to the model’s predictions at every stage of patient’s stay at the hospital.

**Clustering of Phenotypes:** Based on the personalized and data-driven explanations of the predictive ML models at different stages, we present an unsupervised clustering approach, which enables the identification of distinct patient phenotypes within the temporal development of POD, leading to gaining a better understanding, and allowing tailored and more personalized interventions of POD.

## 2 Related work

The combination of explainable machine learning with clustering for patient phenotyping has emerged as a promising paradigm, yet prior work has not addressed longitudinal, multi-stage clinical trajectories. We review three relevant research streams: SHAP-based clustering approaches, the predict-then-cluster paradigm, and foundational critical illness phenotyping methods.

### 2.1 SHAP-based clustering for patient stratification

The approach of clustering patients in SHAP explanation space rather than raw feature space was introduced by Cooper et al. [30], who applied this framework to COVID-19 symptom phenotyping. Cooper et al. [30] proposed to first train an XGBoost classifier, to compute SHAP values, to embed via UMAP, and to cluster with HDBSCAN. It demonstrated that SHAP-based clustering yields more interpretable and well-separated subgroups compared to raw feature clustering. The key insight is that SHAP values rescale heterogeneous features to common units (log-odds contributions) while weighting by predictive importance, effectively de-noising irrelevant features.

Subsequent work has extended this methodology to diverse clinical domains. Rodríguez-Belenguer et al. [31] applied PCA to SVM-derived SHAP values for hematological malignancy phenotyping, identifying patient groups with dramatically different COVID-19 vaccine response profiles. Arslan, et al. [28] demonstrated that clustering on SHAP-transformed data better separates meaningful outcome classes compared to clustering on raw features. More recently, semi-supervised extensions have shown that SHAP-based clustering achieves robust phenotype identification even with limited labeled data [32]. However, these studies share a critical limitation: they focus exclusively on static, cross-sectional patient data, discarding temporal dynamics that may be essential for understanding disease progression.

#### 2.2 Predict-then-cluster vs. cluster-then-predict paradigms.

Conventionally, researchers have used unsupervised clustering to discover subgroups before applying classification—a cluster-first strategy employed to refine disease categorizations [20,21]. While such data-driven groupings can yield valuable insights, a key drawback is that they are agnostic to specific outcomes or labels; the resulting clusters may not correspond to clinically relevant categories and typically require additional interpretation by domain experts [27].

Recent theoretical and empirical work supports the superiority of outcome-driven approaches. Huang et al. [16] formalized this insight in their Deep Significance Clustering (DICE) framework, demonstrating that jointly optimizing representation learning, clustering, and outcome prediction yields subgroups with both statistical significance and predictive utility. Lee and van der Schaar [24] articulated the philosophical shift: patients should be grouped based on similarity of future outcomes rather than solely on similarity of observations. Their Actor-Critic Temporal Predictive Clustering approach uses dynamic cluster assignment as new observations arrive, though it lacks SHAP-based explainability.

Our approach integrates prior knowledge of class labels into the clustering process by clustering in the SHAP-derived feature space. This ensures that groups are directly linked to how predictions are made, highlighting different profiles of feature importance among patients. This supervised-first-then-cluster methodology provides a refined lens to identify subgroups that matter for the prediction task [29].

## 3 Materials and methods

### 3.1 Methodology

#### 3.1.1 Hypothesis of multiple phenotypes in clinical manifestation.

We begin our exploration by considering a cohort represented as ${𝒞}_{N}=\{x_{i},y_{i}{\}}_{i=1}^{N}$, where N denotes the number of patients, $x_{i}\in {\mathbb{R}}^{D}$ denotes the set of features and $y_{i}\in \{0,1\}$ represents the associated labels within a clinical manifestation context, where $y_{i}=1$ represents a positive manifestation. Within this cohort, we hypothesize the existence of multiple phenotypes that are not directly observable in clinical practice. To demonstrate our hypothesis, we will generate synthetic data for which we know the ground truth. In particular, we categorize the features of $x_{i}$ into three distinct types: shared, informative, and noisy, as denoted as $x_{i}=\{x_{i}^{\text{shared}},x_{i}^{\text{informative}},x_{i}^{\text{noisy}}\}$. Here, shared features refer to those common across all phenotypes, whereas informative features are unique and predominant within specific phenotypes, playing a crucial role in their differentiation. Both shared and informative features are instrumental in determining the phenotypes, while noisy features represent extraneous information that does not contribute to phenotype identification. This conceptualization allows us to define a phenotype through a binary-valued function depending on its shared and informative features, denoted as:


$$\begin{array}{c} \begin{array}{cc} {\text{ph}}_{i,\alpha } & =f_{\alpha }(x_{i,\alpha }^{\text{informative}})\\ {\text{ph}}_{i,\beta } & =f_{\beta }(x_{i}^{\text{shared}},x_{i,\beta }^{\text{informative}})\\ {\text{ph}}_{i,\gamma } & =f_{\gamma }(x_{i}^{\text{shared}},x_{i,\gamma }^{\text{informative}})\\ {\text{ph}}_{i,\delta } & =f_{\delta }(x_{i}^{\text{shared}},x_{i,\delta }^{\text{informative}}) \end{array}\; \end{array}$$ (1)

where function *f* should return true if the data point belongs to the phenotype, and false otherwise.

Correspondingly, phenotype labels within manifestation *y* can be classified as follows:


$$\begin{array}{c} y_{i}=\left\{ \begin{array}{cc} 0 & \text{if }{\text{ph}}_{i,\alpha },\\ 1 & \text{if }{\text{ph}}_{i,\beta }\text{ or }{\text{ph}}_{i,\gamma }\text{ or }{\text{ph}}_{i,\delta }, \end{array}\right.\; \end{array}$$

where the labels are determined by the presence of specific phenotype-defining features. In Sect 3.2.1 we will provide specific choices for these phenotype functions in Equation (1).

#### 3.1.2 Pipeline: Predictive-clustering algorithm generalizable to phenotypes.

**Fig 1:**
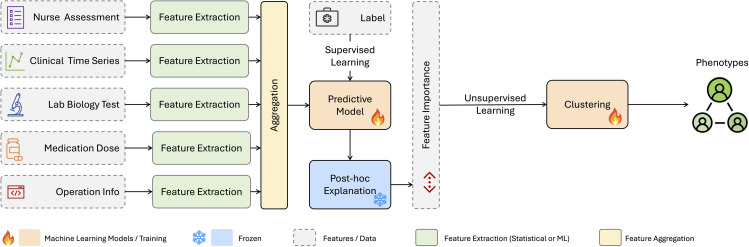
Predictive-Clustering Algorithm Generalizable to Phenotypes.

To test our hypothesis, we develop a simple algorithm that can be generalized to any clinical phenotype identification process. The algorithm consists of three main steps: training a predictive model, performing post-hoc analysis using SHAP, and conducting phenotype clustering based on SHAP values (Fig 1). The following outlines the detailed design and sequential steps of the method:

**Training a predictive model:** By utilizing only the binary labels $y_{i}$, we train a prediction model to estimate the probability of a patient having the disease. Our goal is to learn the conditional expectation


$$\mu (x)=𝔼[y_{i}|x_{i}=x],$$

where $x_{i}$ represents the feature set for patient *i*.

**Compute Personalized Explanations:** After training the prediction model, we perform a post-hoc analysis to determine the level of importance of all characteristics to get personalized explanations. We employ the SHapley Additive exPlanations (SHAP) algorithm for this purpose. SHAP is a game-theoretic approach that assigns each feature an importance value, known as the SHAP value, which represents the feature’s contribution to the model’s prediction. The SHAP values provide an agnostic measure of feature importance across different models that can be used to interpret the model’s behavior. Please note that additional explainability techniques, like integrated gradients [33], can also be incorporated into our workflow.

**Phenotype clustering based on SHAP Explanations:** Using the personalized explanations (SHAP values) obtained from the post-hoc analysis, we perform phenotype clustering. By clustering patients based on their personalized explanations, we aim to identify distinct phenotypes *within* the cohort [34]. The clustering algorithm groups patients with similar SHAP value patterns, indicating that they share common important features that contribute to their phenotype. This step allows us to find different explanations in the development of POD and to uncover potential subtypes or phenotypes within the disease cohort, providing a more granular understanding of the disease heterogeneity.

By combining predictive modeling, post-hoc analysis, and phenotype clustering, our algorithm offers a generalizable and actionable approach to identifying clinical phenotypes. This methodology can be applied to various clinical diseases and can aid in the discovery of meaningful patient subgroups, leading to more targeted and personalized treatment strategies.

#### 3.1.3 Notation of peri-operative delirium case.

**Fig 2:**
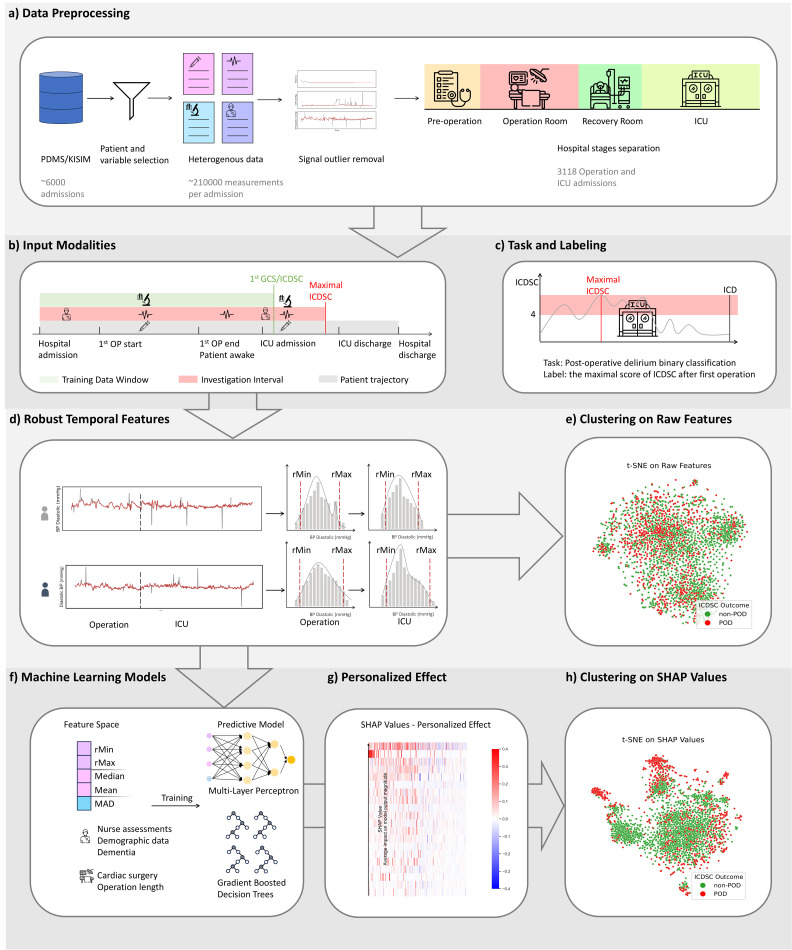
Comprehensive workflow for analyzing the development of peri-operative delirium based on multi-modal disease trajectories using explainable ML for data-driven phenotype clustering.

We consider longitudinal data ${𝒟}_{N}=\{X_{i},Y_{i}{\}}_{i=1}^{N}$ with input $X_{i}$ and outcome $Y_{i}$ time series, respectively, from *N* patients. The temporal input data involve the multivariate time series $X_{i}=[x_{i,t}{]}_{t=1}^{T}\in 𝒳={\mathbb{R}}^{T\times D}$, where $x_{i,t}=[x_{i,t}^{d}{]}_{d=1}^{D}\in {\mathbb{R}}^{D}$. These input data consist of complex and temporal multi-modal data from different sources, such as demographic information, clinical nurse assessments, operation details, biometric monitoring signals, laboratory test results, medication dosages, and blood gas analyses. Further, we consider a binary outcome time series $Y_{i}=[y_{i,s}{]}_{s=T}^{S}\in 𝒴=\{0,1{\}}^{S-T}$. These labels correspond to a few temporal assessments indicating the physiological state of the patient such as ICDSC [35] for ICU delirium. We focus on the setting, where the observed times of the outcomes are non-overlapping with the input time series, that is, we have s≥T. We want to emphasize that this is a particularly challenging setting as we only observe labels far in the future. Following the data processing pipeline in Fig 2, we introduce some specific time points to split the input time series into clinically meaningful periods, such as pre -OP, intra -OP, and post -OP. For instance, we have $\tau_{\text{pre}}=[\tau_{\text{pre}}^{0},\tau_{\text{pre}}^{1}]$ indicating the starting $\tau_{\text{pre}}^{0}$ and ending $\tau_{\text{pre}}^{1}$ time of the preoperative period, respectively. Consequently, we refer to $X_{i,\text{pre}}=[x_{i,t}{]}_{t=\tau_{\text{pre}}^{0}}^{\tau_{\text{pre}}^{1}}$ for the preoperative input time series. Similarly, we define $\tau_{\text{intra}}$ and $\tau_{\text{post}}$, leading to the intraoperative $X_{i,\text{intra}}$, and postoperative time series $X_{i,\text{post}}$ of patient *i*. Moreover, we introduce the corresponding *cumulative* input time series $X_{i,{\text{intra}}^{+}}=[x_{i,t}{]}_{t=0}^{\tau_{\text{intra}}^{1}}$, which always start from t=0 and include time points up to the ending time point of the particular period $\tau_{\text{intra}}^{1}$. For the sake of simplicity, we omit the explicit dependency on *i* when it is clear from the context, and use for instance $X_{i}=X$ and $Y_{i}=Y$, respectively.

#### 3.1.4 Our prediction goal for peri-operative delirium.

We want to estimate the probability of the temporal outcomes $Y_{s}$ at any time point k<s≤S given time-varying inputs $X_{t:k}$ from the time period *t* up to *k*. In particular, we want to learn the conditional expectation


$$\mu_{t:k}^{s}(x)=𝔼[Y_{s}|X_{t:k}=x],$$

where $\mu_{t:k}^{s}:{\mathbb{R}}^{(k-ttimesD}\to \{0,1\})$ maps the different temporal inputs to the binary outcome. For instance, we consider the stage-wise independent predictions


$$\mu_{\text{intra}}^{s}(x)=𝔼[Y_{s}|X_{\text{intra}}=x]$$

with $t=\tau_{\text{intra}}^{0}$ and $k=\tau_{\text{intra}}^{1}$. Further, for the cumulative predictions, we aim to compute


$$\mu_{{\text{intra}}^{+}}^{s}(x)=𝔼[Y_{s}|X_{{\text{intra}}^{+}}=x],$$

with t=0 and $k=\tau_{\text{intra}}^{1}$. Similarly, for the pre- and postoperative time periods, the goal is to compute $\mu_{\text{pre}}^{s}(x)$, $\mu_{{\text{pre}}^{+}}^{s}(x)$, $\mu_{\text{post}}^{s}(x)$, and $\mu_{{\text{post}}^{+}}^{s}(x)$.

#### 3.1.5 Abstracting temporal complexity in real-world clinical time series.

Since it is challenging to learn the full temporal distributions $\mu_{t:k}^{k:S}(x)=𝔼[Y_{k:S}|X_{t:k}=x]$ from noisy real-world longitudinal patient data [36] with many missing values, weak signals, and complex temporal modalities, we follow a pragmatic approach by projecting the input and outcome time series by collapsing the temporal ordering with specified mappings $\psi :\{0,1{\}}^{S-k}\to \{0,1\}$ and $\phi :{\mathbb{R}}^{(k-t)\times D}\to {\mathbb{R}}^{M\times D}$, with M≪(k−t). In particular, we consider a simplified conditional expectation


$$\mu_{t:k}(x)=𝔼[\psi \left(Y_{k:S}\right)|\phi \left(X_{t:k}\right)=x],$$ (2)

with $\mu_{t:k}:{\mathbb{R}}^{M\times D}\to \{0,1\}$. Besides practical reasons such as robustness against overfitting and noise in the data, this can be further justified in our context since during a certain time period, e.g., during an operation, the specific time of a certain event is often not relevant, instead, the overall distribution over time is more important.

Recognizing that raw time series can be less explainable due to their intricate fluctuations and volume of data points, we turn to distribution-based abstraction guided by clinical knowledge to extract information from time series data. This approach is motivated by several considerations. First, clinical time series data is inherently sparse and irregularly sampled, containing substantial noise that can lead models to learn spurious patterns and overfit to random fluctuations rather than meaningful clinical signals. By transforming temporal segments into statistical distributions, we effectively smooth out measurement noise and handle missing data points, creating more stable representations that are less susceptible to overfitting on sparse, noisy observations. Second, this approach aligns with established clinical practice, where physicians routinely examine statistical summaries of temporal measurements (e.g., mean blood pressure over 24 hours, glucose variability metrics). By using distribution-based features such as mean, median, and percentiles, our model’s decision-making process becomes more interpretable to clinicians, as it mirrors their natural reasoning patterns, thereby enhancing trust and facilitating clinical adoption. Furthermore, extracting features from temporal distributions is particularly advantageous for harnessing the full potential of diverse data modalities in clinical EHR datasets.

For example, the overall fraction that specific variables are in a certain range, i.e., above a certain value, is often more clinically relevant than the exact values during that time. For instance, we define hyperoxemia as an SpO_2_ level exceeding 98%, and when the SpO_2_ level reaches 100%, it is considered severe hyperoxemia. Hence, we use different quantities to describe the temporal input distributions, that is, we use


$$\phi \left(X_{t:k}\right)=[x_{\text{rMin}},x_{\text{rMax}},x_{\text{Median}},x_{\text{Mean}},x_{\text{MAD}}]$$

for the inputs, where rMin  and rMax  correspond to the 5% and 95% quantiles, respectively, and MAD  to the *median absolute deviation*. These statistics were mainly guided by domain expert (clinicians and physicians). For the outcome, we use the maximum of the observed labels, that is,


$$\psi \left(Y_{T:S}\right)=\max(Y_{T:S}),$$

describing the most severe event in the postoperative time span. Therefore, we aim to learn the three stage-wise independent conditional expectations $\mu_{\text{pre}}(x)$, $\mu_{\text{intra}}(x)$, and $\mu_{\text{post}}(x)$, as well as the cumulative $\mu_{{\text{pre}}^{+}}(x)$, $\mu_{{\text{intra}}^{+}}(x)$, and $\mu_{{\text{post}}^{+}}(x)$. Those are obtained by plugging-in the corresponding inputs, for instance we have $\mu_{\text{intra}}(x)=𝔼[\psi \left(Y_{T:S}\right)|\phi \left(X_{\text{intra}}\right)=x]$ for $X_{\text{intra}}$.

#### 3.1.6 Prediction models.

For learning the conditional expectation in (2) $\mu_{t:k}(xcolon{\mathbb{R}}^{M\times D}\to \{0,1\})$, we use different ML classification models to get an estimate


$${\hat{\mu }}_{t:k}(x):{\mathbb{R}}^{M\times D}\to \{0,1\}.$$ (3)

In particular, we consider the three stage-wise independent models ${\hat{\mu }}_{\text{pre}}(x)$, ${\hat{\mu }}_{\text{intra}}(x)$, ${\hat{\mu }}_{\text{post}}(x)$ as well as the cumulative ${\hat{\mu }}_{{\text{pre}}^{+}}(x)$, ${\hat{\mu }}_{{\text{intra}}^{+}}(x)$, ${\hat{\mu }}_{{\text{post}}^{+}}(x)$. We train five different machine learning models for each prediction task: logistic regression [37], multilayer perception (MLP) [38,39], random forest [40], gradient boosting [41], and extreme gradient boosting (XGBoost) [42].

#### 3.1.7 Personalized explainability.

The learned ML classification models ${\hat{\mu }}_{t:k}(xcolon{\mathbb{R}}^{M\times D}\to \{0,1\})$ in (3) can be used for analysing the influence of the input features $x\in {\mathbb{R}}^{M\times D}$. In particular, for a given trained ML model, we can compute the SHAP values for each input feature $x^{m,d}$, that is,


$$\theta^{m,d}(x)=𝔼\left[\hat{\mu }(X)|X^{m,d}=x^{m,d}\right]-𝔼\left[\hat{\mu }(X)\right],$$

indicating the difference between the expected baseline $𝔼\left[\hat{\mu }(X)\right]$ and the expected outcome when changing feature $X^{m,d}=x^{m,d}$ (i.e., $𝔼\left[\hat{\mu }(X)|X^{m,d}=x^{m,d}\right]$), for which we refer to [17–19]. In particular, we consider the *personalized* SHAP value


$$\theta_{i}^{m,d}(x_{i}):{\mathbb{R}}^{M\times D}\to \mathbb{R}$$

when using the inputs $x_{i}\in {\mathbb{R}}^{M\times D}$ of patient *i*. Therefore, we can define the complete personalized explanation $\theta_{i}=[\theta_{i}^{m,d}{]}_{m=M,d=D}\in {\mathbb{R}}^{MD}$ and further all personalized values of a cohort $\Theta ={\left[\theta_{i}\right]}_{i=N}\in {\mathbb{R}}^{MD\times N}$ as illustrated in Fig 5. Note that we can compute personalized explanations for different $\Theta_{t:k}$ corresponding to the trained ML classifier ${\hat{\mu }}_{t:k}(x)$ in the time-period between *t* and *k*.

**Fig 3:**
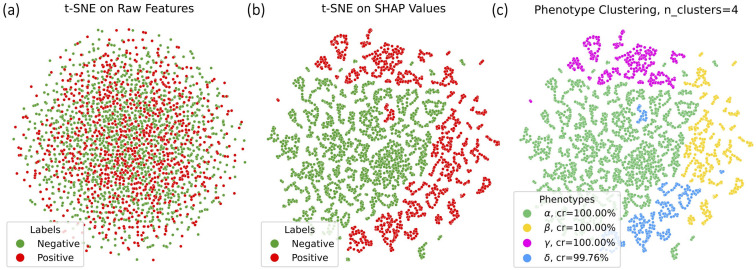
T-SNE visualization of (a) raw features, (b) SHAP values and the ground truth outcomes for POD, and (c) SHAP [17] values with ground truth phenotype labels.

**Fig 4:**
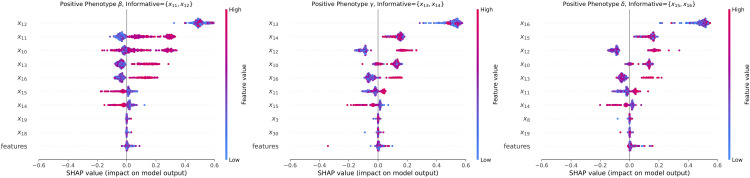
SHAP. [17] analysis for different phenotypes within the cohorts where $y_{i}=1$.

#### 3.1.8 Clustering of explainability space.

We can use the personalized SHAP (i.e., feature importance) $\theta_{i}^{m,d}$ summarized in $\Theta \in {\mathbb{R}}^{MD\times N}$ to answer data-driven question whether there are different phenotypes *explaining* the development of delirium. In particular, we aim to find *K* subgroups in an unsupervised manner based on the personalized explanations which are important in distinguishing whether or not the patient develops postoperative delirium. This enables us to train a clustering algorithm on the personalized explanations ${\left[\theta_{i}\right]}_{i=N_{train}}\in {\mathbb{R}}^{MD\times N_{train}}$ in the training set yielding a clustering function


$$\Pi (\theta ):{\mathbb{R}}^{MD}\to \{1,\ldots ,K\}$$

for a patient’s explanation $\theta \in {\mathbb{R}}^{MD}$. More specifically, we can define the temporal clusters $\Pi_{t:k}(\theta_{t:k})$ based on the personalized explanations $\Theta_{t:k}$ corresponding to the trained ML classifier ${\hat{\mu }}_{t:k}(x)$. Note that this clustering algorithm is rather different from a clustering algorithm trained on raw inputs $x\in {\mathbb{R}}^{M\times D}$ or raw temporal inputs $x\in {\mathbb{R}}^{(k-ttimesD})$. More importantly, clustering using personalized SHAP scores is more robust (as we will show) as it finds different clusters/subgroups in the explanations of the learned mapping of inputs to delirium labels, allowing to draw data-driven hypotheses for unsupervised phenotypes. By applying hierarchical clustering [43] to the personalized SHAP scores, our proposed method is capable of finding meaningful phenotypes with both synthetic and real-world delirium data, as demonstrated later in Sect 4.

### 3.2 Dataset

#### 3.2.1 Synthetic dataset.

In this section, we focus on the generation of a synthetic dataset involving $X\in {\mathbb{R}}^{N\times D}$ and $y\in {\mathbb{R}}^{N}$, where *N* denotes the number of samples and $D=D^{\text{shared}}+D^{\text{informative}}+D^{\text{noisy}}$ represents the number of features, as outlined in Sect 3.1.1. We assume that the *D* features can represent any kind of clinical data, including clinical time series, EHR data, or multi-omics data. The main purpose of the generation of the synthetic data is to compare the ground truth phenotype with the discovered phenotype as predicted by our workflow algorithm, as this can never be evaluated on real-world data.

Within the input feature space, we identify a critical subset of $D^{\text{informative}}$ for each phenotype. We define a simple structural equation model (SEM) [44] so that the informative features significantly influence the phenotype labels ${\text{ph}}_{i,z}\in \{0,1\}$ in Equation (1) for all phenotypes *z* and patients *i*. To mimic delirium phenotypes in our synthetically generated data, we employ the predefined set of informative features as the basis for generating them where $y_{i}=1$, as previously hypothesized in Sect 3.1.1. Each sample is assigned to a phenotype based on its randomly generated feature profile. This design approach is driven by the complex, multi-causal aspects of postoperative delirium, focusing on identifying predictive clinical indicators within a huge dataset.

In our synthetic experiment, we generate a dataset comprising N=3000 samples with D=30 features, where each feature $x_{d}$ (d=1,…,D) is independently sampled from a standard Gaussian distribution 𝒩(0,1). The dataset incorporates multiple phenotypes: one phenotype (*α*) represents the negative class (to mimic non-delirium cohort), while three phenotypes (*β*, *γ*, and *δ*) characterize the positive class (to mimic delirium cohort). Each positive phenotype is defined by $D^{\text{informative}}=2$ informative features, with $D^{\text{shared}}=1$ feature shared among the positive phenotypes. For the negative class (phenotype *α*), we designate three distinct informative features that do not overlap with those used in the positive phenotypes. The cohort assignment criteria are outlined in the Algorithm 1.

**Algorithm 1. Phenotype Assignment Criteria in a Python-like Style.**

import numpy as np

def f_alpha(x1, x2, x3):

 # Conditions for f_alpha phenotype

 return np.logical_and(x1 < 0, np.logical_and(x2 < 0, x3 < 0))

def f_beta(x10, x11, x12):

 # Conditions for f_beta phenotype

 return np.logical_and(np.logical_or(x10 > 0.5, x11 > 0.5), x12 > 0.5)

def f_gamma(x10, x13, x14):

 # Conditions for f_gamma phenotype

 return np.logical_and(x10 <= 0.5, np.logical_and(x13 > 0.5, x14 <= 0.5))

def f_delta(x10, x15, x16):

 # Conditions for f_delta phenotype

 return np.logical_and(x10 <= 0.5, np.logical_and(x15 <= 0.5, x16 > 0.5))

#### 3.2.2 Peri-operative delirium dataset.

We use a dataset consisting of multi-modal and temporal electronic health record (EHR) data (Fig 2a-b) from patients admitted to the Intensive Care Unit (ICU) of the local University Hospital between the year of 2017 and 2022. The data covers pre-, intra-, and postoperative stages, offering a comprehensive view of each patient’s health journey. The dataset includes vital signs, laboratory test results, medication history, demographic details, and operation-related information. Patients aged 65 years or older were included in the study and classified as cases (those who developed and received treatment for delirium during their ICU stay) or controls (those who did not meet the delirium criteria). To ensure data quality and ethical compliance, patients who objected to the use of their personal health data for research or had an ICU stay shorter than 24 hours were excluded. In this study, we utilize the Intensive Care Delirium Screening Checklist (ICDSC) [35] as the primary measure for identifying POD. The ICDSC is among the most developed and validated tools for this purpose, offering a comprehensive framework to systematically assess and diagnose delirium in a clinical setting [45]. We define a patient as being in delirium if the ICDSC score exceeds 3 at any point during a week-long ICU stay. A detailed description of the data and their processing methodologies is provided in the supporting information S1 Text.

## 4 Results and discussion

### 4.1 Experimental settings

In our study, we evaluate the prediction models using both the Area Under the Receiver Operating Characteristic (AUROC) and the Area Under the Precision-Recall Curve (AUPRC), which capture the model’s overall discrimination capability and its precision-recall trade-off, respectively. The final performance is computed as the average over test sets from 10 independent train-test splits. For a detailed description of the experimental settings, please refer to the supporting information S2 Text.

### 4.2 Synthetic dataset

#### 4.2.1 Analysis of clustering challenges in raw feature space.

As a comparison, we first try to cluster the raw features of the synthetic data to identify phenotypes. However, as shown in Fig 3, the clustering algorithm was unable to effectively distinguish between different phenotypes in the raw feature space. This suggests that the raw features may contain a lot of noise, making it challenging for the clustering algorithm to directly discover phenotype characteristics.

#### 4.2.2 Phenotype clustering outcomes.

We then trained a classifier to predict the outcome label *y*, followed by computing SHAP values from the trained model, and then performing hierarchical clustering [43] using the computed SHAP values. To evaluate their clustering effectiveness, we define the correct rate (**cr**) as dividing the number of samples that are accurately categorized according to their actual phenotype by the overall number of samples within the cluster, following the approach described in [16]. This rate measures the accuracy with which samples are assigned to their true categories within a given cluster. As illustrated in Fig 3 (c), the clustering algorithm was able to identify phenotypes within the feature-importance space (**cr** rate is equal or close to 100% for all phenotypes). This suggests that the SHAP values can capture the important features and their contributions to the classifier’s predictions, enabling the discovery of phenotype-specific patterns.

#### 4.2.3 SHAP analysis to prove our hypothesis.

Given that the most important features for different phenotypes vary and consist of both informative features specific to each phenotype and shared ones common across all of them, we wanted to see if this can be identified using our proposed workflow, specifically the analysis of SHAP values and ranking important features. The results, as shown in Fig 4, demonstrate that the top important features (using SHAP scores) overlap with the ground-truth top important features for each phenotype, with a combination of phenotype-specific informative and shared features. This finding supports our proposed workflow of using clustering approach on SHAP values instead of raw input features.

In summary, our analysis reveals that clustering in the raw feature space is challenging due to potential noise and the complexity of phenotype characteristics. By training a predictive model and leveraging SHAP values, we were able to effectively identify phenotypes and validate our hypothesis regarding the importance of phenotype-specific and shared features. These findings provide valuable insights into the underlying patterns and characteristics of different phenotypes in our synthetic data.

### 4.3 Peri-operative delirium dataset

#### 4.3.1 Data description.

Using the preprocessing pipeline shown in the supporting information **S1.2.1** in S1 Text, our final cohort consists of 3,118 patients, each with 680 statistical features initially considered. To ensure data quality, we excluded features for which more than 50% of patients did not have a recorded value, resulting in a final set of 587 statistical features for analysis.

#### 4.3.2 Prediction results and personalized risk factor explanations.

**Fig 5:**
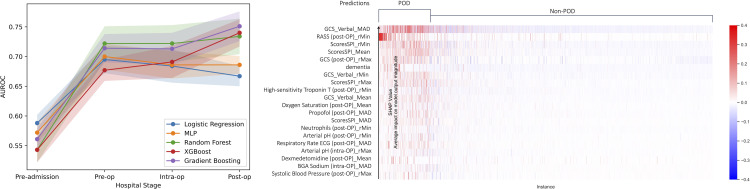
Predictive model performance for different hospital stages and personalized explanation with cohort with post^+^-op features.

Following the pipeline described in Sect 3.1.2 and the methods in Sect 3.1.4, we apply different predictive machine learning models described in Sect 3.1.6 for different hospital stages. In the comparative analysis of predictive models for POD, Gradient Boosting has the highest overall performance across all models during every hospital stage, reflecting its capacity to model diverse data modalities and stages of hospital care. The comprehensive utilization of data spanning across all hospital stages yielded optimal performance of AUROC 0.743±0.019 and AUPRC 0.533±0.029. When considering only intra-operative data, a marginal decrease in predictive accuracy is observed for trained models. This could suggest the presence of noise within intra-operative patient data. Moreover, the cumulative models trained on all previous history showed consistently better performance compared to non cumulative counterparts (i.e., ones trained on start and end of each stage - see Table 1). Overall, Gradient Boosting model achieved a consistent improving performance, across all stages, showcasing its robustness in modeling multi-modal data including the noisy interval of intra-operative phase (Fig 5 left panel).

**Table 1:** Models Performance for different hospital stages.

Models	pre^+^-OP		intra^+^-OP		post^+^-OP	
	AUROC	AUPRC	AUROC	AUPRC	AUROC	AUPRC
Logistic Regression	0.698±0.023	0.454±0.022	0.666±0.029	0.427±0.035	0.659±0.023	0.428±0.033
Multilayer Perceptron	0.704±0.029	0.452±0.030	0.669±0.030	0.429±0.029	0.667±0.025	0.433±0.035
Random Forest	0.721±0.029	0.499±0.037	0.712±0.034	0.474±0.036	0.724±0.031	0.490±0.037
XGBoost	0.675±0.014	0.445±0.019	0.690±0.024	0.454±0.033	0.731±0.021	0.512±0.028
Gradient Boosting	0.714±0.026	0.485±0.029	0.704±0.027	0.466±0.029	0.743±0.019	0.533±0.029
**Models**	**pre-OP**		**intra-OP**		**post-OP**	
	**AUROC**	**AUPRC**	**AUROC**	**AUPRC**	**AUROC**	**AUPRC**
Logistic Regression	0.691±0.024	0.426±0.022	0.561±0.029	0.322±0.026	0.592±0.018	0.360±0.016
Multilayer Perceptron	0.696±0.029	0.436±0.029	0.551±0.030	0.318±0.026	0.598±0.030	0.362±0.026
Random Forest	0.719±0.029	0.498±0.037	0.576±0.026	0.326±0.026	0.653±0.030	0.419±0.041
XGBoost	0.680±0.019	0.449±0.019	0.556±0.019	0.316±0.015	0.652±0.024	0.426±0.021
Gradient Boosting	0.714±0.026	0.474±0.026	0.558±0.019	0.319±0.018	0.674±0.025	0.462±0.029

We employ personalized SHAP scores as described in Sect 3.1.7 to elucidate the attribution of various features in the model prediction. This approach of personalized explanation allows us to further explore each individual’s case, providing a more detailed understanding of the diverse factors influencing their health outcomes. By examining the specific contributions of different features to the risk of developing delirium for each patient, we observe that the top 5 risk factors come from clinical assessments, including GCS (Glasgow Coma Scale) [46], RASS (Richmond Agitation-Sedation Scale) [47], and SPI (Self-Care Index, German: Selbstpflege-Index) [48], as shown in Fig 5 (right panel). Variable risk factors among different individuals provide insights into their unique clinical conditions. Furthermore, this proposed workflow helps identify different cohorts of patients with respect to the causes of delirium, enabling targeted interventions and more effective management strategies tailored to the unique risk profiles of different groups.

#### 4.3.3 Analysis of clustering in the raw feature space.

**Fig 6:**
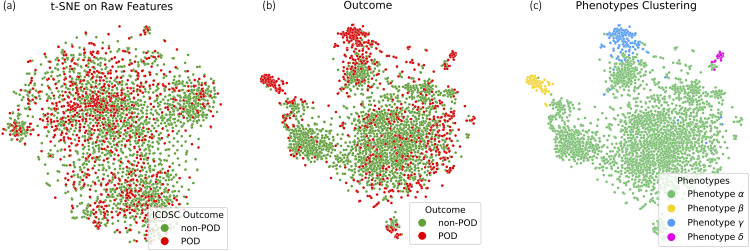
T-SNE visualization of a) raw features, b) SHAP values and the ground truth outcomes for POD, and c) SHAP values with assigned phenotype clusters.

As a comparison, we first attempted to cluster the raw features of the real delirium data to identify the phenotypes. However, as shown in Fig 6(a) with more results with different dimension reduction algorithms in Appendix Fig. **S2**, the clustering algorithm was unable to effectively distinguish between different phenotypes in the raw feature space. The results show that there is no clear separation based on raw features alone, indicating that relying solely on raw features is insufficient to discover delirium phenotypes. This suggests that raw features may contain noise and complex interactions as we hypothesized, making it challenging for the clustering algorithm to directly uncover the characteristics of the phenotype.

#### 4.3.4 Identifying patient subgroups through clustering in feature-importance space.

To further investigate the phenotype characteristics within the delirium data, following the algorithm described in Sect 3.1.2, we computed SHAP values on the trained Gradient Boosting classifier and applied hierarchical clustering on the SHAP values. We set the number of cluster as four since it showed a good trade-off based on quantitative measurement as shown in the supporting information **S3.2** in S3 Text and clinical guidance by the collaborating physicians. As illustrated in Fig 6(b) and (c), the clustering algorithm was able to identify distinct patient subgroups within the feature-importance space. The t-SNE visualization of the SHAP values shows clear separations between the identified clusters, indicating that the SHAP values capture the important features and their contributions to the classifier’s predictions, enabling the discovery of phenotype-specific patterns and explanations.

#### 4.3.5 Phenotype-based explainability analysis.

**Fig 7:**
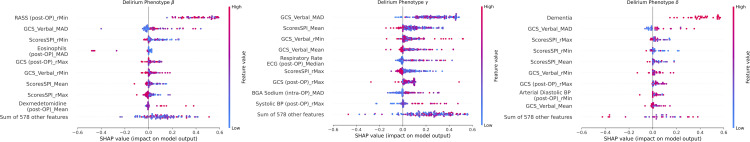
SHAP analysis for different phenotypes within delirium cohorts.

In the analysis of phenotype characteristics using SHAP, our study has identified distinct risk factors that play a dominant role in the manifestation of delirium across different phenotypes. This underscores the clinical diversity observed in delirium presence and suggests that each phenotype may be driven by unique underlying mechanisms or pathways [2]. For instance, as shown in Fig 7, in phenotype *β*, we can find that low eosinophil count [49] is one of the risk factors of POD and the use of dexmedetomidine [50] is correlated to reduced POD. The prominence of eosinophil count [51,52] suggests a potential link between immune system dysfunction and the development of delirium. Additionally, the identification of dexmedetomidine as a significant factor indicates that the choice of sedative agents may have a significant impact on the risk of delirium in this subgroup. Please note that there are some cases where the usage of dexmedetomidine will develop POD. We aim to investigate the multi-causal factors contributing to these cases in the future work. In phenotype *γ*, the respiratory rate ECG and systolic blood pressure [53,54] in the ICU room are more important for the development of delirium. This suggests that phenotype *γ* may be more associated with cardiovascular and respiratory dysfunction, potentially indicating a greater influence of physiological stressors on the development of delirium in this subgroup. A particularly notable finding is the strong association between dementia and delirium in phenotype *δ*. In this group, dementia was a common condition among all patients, marking it as a key risk factor. This observation is critical because the correlation between dementia and POD has long been established, as evidenced by results from the National Inpatient Sample (NIS) database, where dementia patients had a higher POD (15.4% vs 1.5%, p=<0.001) as compared with patients with no dementia [55]. Therefore, this finding strongly supports the validity of our approach.

**Fig 8:**
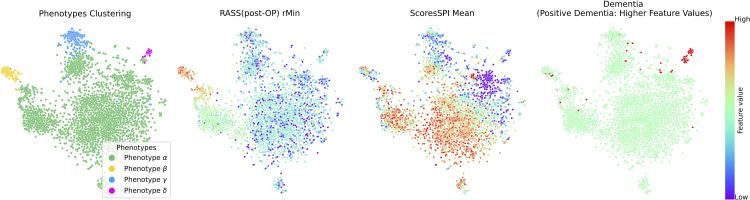
Bridging SHAP analysis and clinical raw feature interpretation.

We then sought to connect these interpretable insights back to the original raw features from which they were derived. As shown in Fig 8, based on the SHAP analysis, we identified three clinical features that influence the occurrence of delirium. A higher robust minimal value of RASS can be related to a higher incidence of delirium in phenotype *β*. The lower mean value of the SPI score is probably associated with a higher incidence of delirium in the phenotype *γ*. As expected, in the phenotype *δ*, almost all patients have dementia.

#### 4.3.6 Temporal evolution of SHAP-based clusters.

**Fig 9:**
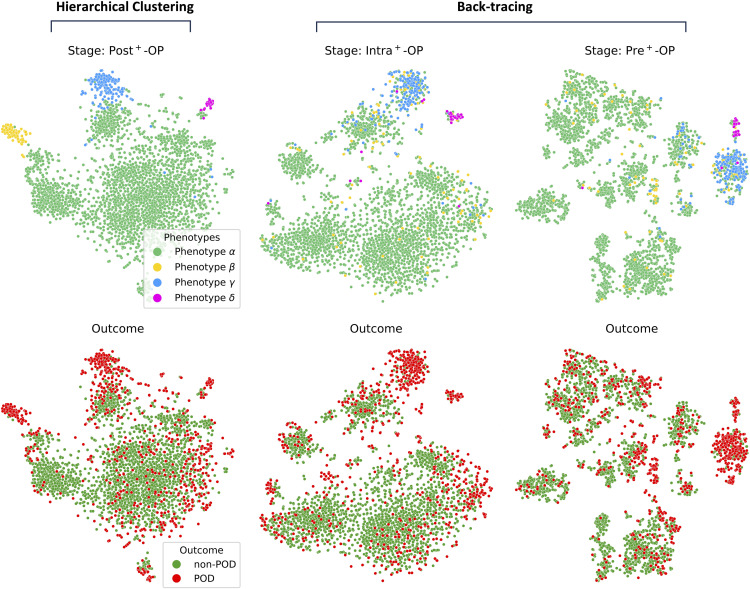
Development of subgroups through different hospital stages.

Subsequently, we focused on clustering the SHAP features across different hospital stages. Fig 9 illustrates that the **phenotype *γ*** is more distinctly identifiable, indicating a unique set of risk factors that can be recognized early on. In contrast, **phenotype *β*** and **phenotype *δ*** initially appear dispersed within the space. However, as patients progress through the various stages of hospital care, these two subgroups gradually become more defined and separate from each other. This evolution highlights the dynamic nature of POD risk as influenced by the changing clinical landscape, demonstrating that certain risk factors become more or less prominent as the patient’s condition evolves.

#### 4.3.7 Phenotype clustering stability analysis.

**Fig 10:**
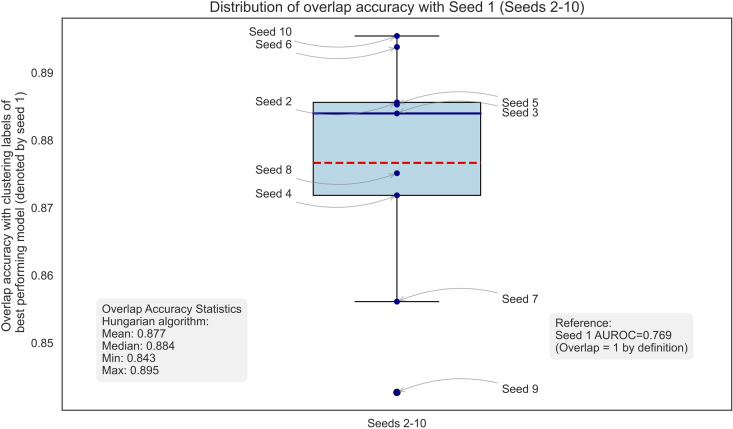
Overlap accuracy with clustering labels of best performing model (denoted by seed 1).

We further tested the stability of our clustering results by repeating the same clustering analysis using the other nine models trained with different seeds (we had 10 independent model training with different random seeds and train-validation splits). We compared the resulting phenotype assignments (i.e., assignment of 4 clusters) across these variations with the assignment of the best-performing model (denoted by seed 1) that we report in this paper. We did this for all data points and then calculated the overlap accuracy between the cluster assignments of each of the nine models and our best performing model (i.e., seed 1) after optimally matching these assignments using the Hungarian algorithm [56,57]. The results show substantial consistency in the identification of the phenotype (that is, clustering the data) despite variations in model training and data partitioning, with a median overlap accuracy greater than 88%, as shown in Fig 10.

## 5 Limitation and future work

Regardless of how sophisticated a model may be, data quality remains a fundamental bottleneck in clinical time series analysis. In our study, we encountered substantial challenges arising from noisy, sparse, and inconsistent data. Many hospital datasets exhibit a low signal-to-noise ratio: vital signs may be recorded irregularly, key contextual variables are often missing, and documentation practices vary across clinical settings. These issues are further exacerbated by extensive missing values, outliers, and occasional erroneous measurements. Such limitations are well recognized in healthcare, where data are collected under heterogeneous conditions and varying clinical standards. Without careful mitigation, these factors can undermine the performance of even advanced continuous-time models, sometimes leading them to underperform compared to simpler, more robust approaches that rely on stronger assumptions or extensive preprocessing.

Addressing these challenges requires close interdisciplinary collaboration. Clinicians and domain experts play a crucial role in defining principled guidelines for preprocessing raw clinical data, such as establishing criteria for outlier detection, identifying implausible vital sign fluctuations, and determining appropriate strategies for handling missing values. At the same time, computer scientists and data scientists must design and implement scalable, robust algorithms that can operationalize these guidelines in practice [58]. Effective techniques for noise reduction, outlier handling, and imputation should be automated while preserving clinically meaningful signals. By integrating domain-informed preprocessing—potentially as part of the modeling pipeline itself—we can improve data reliability and strengthen the foundation upon which learning algorithms operate. Ultimately, only through sustained clinician–developer collaboration can advanced machine learning methods be deployed on clinical data with confidence.

A limitation of our work is the reliance on accurately labeled outcome data, which are often scarce or costly to obtain. Many complex conditions, including delirium and rare diseases, are difficult to label at scale, posing a significant challenge for supervised learning approaches. One promising direction is augmenting limited real-world data with simulated patient data informed by medical knowledge. For instance, the SHEPHERD framework for rare disease diagnosis was trained primarily on a large cohort of simulated patients generated from known phenotype–disorder associations [59]. By grounding simulation in biomedical prior knowledge and integrating it with a knowledge graph, the model was able to perform tasks such as causal gene discovery and patient similarity analysis despite having few real cases [59]. This work illustrates a broader strategy for the field: leveraging domain knowledge to construct hybrid datasets that combine real and realistic synthetic examples. Future research in clinical time series modeling should explore similar approaches, such as simulating patient trajectories under rare or hypothetical scenarios, to enrich training data. Embedding medical prior knowledge into the learning process in this manner may enable models to generalize more effectively and capture clinically meaningful patterns that are difficult to infer from limited real-world datasets alone.

## Supporting information

S1 Text Data Description for Delirium Case Study. S1 Text describes the data acquisition, preprocessing, and labeling pipeline for a postoperative delirium (POD) prediction study, detailing how heterogeneous EHR data—vitals, labs, medications, and assessments were extracted, cleaned, and structured across pre-, intra-, and postoperative stages to train machine learning models using ICDSC-based delirium labels.
(PDF)

S2 Text Experimental Settings. S2 Text details the experimental setup, including synthetic and clinical dataset protocols, a robust 10-fold cross-validation scheme repeated across 10 random seeds, AUROC/AUPRC evaluation metrics, and computational cost benchmarks for various model–SHAP explainer combinations used in the delirium prediction study.
(PDF)

S3 Text Supplementary Results and Intermediate Findings. S3 Text presents intermediate results including dimensionality reduction visualizations, cluster number selection, risk stratification across hospital stages, phenotype clustering at each perioperative stage, minimized-feature model performance, and an analysis of label noise arising from discrepancies between ICDSC and ICD-based delirium diagnoses.
(PDF)
