# Supplementary material for: Clustering of disease trajectories with explainable machine learning: A case study on postoperative delirium phenotypes
Source: PLOS Digit Health. 2026 Mar 23;5(3):e0001267. doi: 10.1371/journal.pdig.0001267 (PMC13008057; doi:10.1371/journal.pdig.0001267)
Supplement: S1 Text — describes the data acquisition, preprocessing, and labeling pipeline for a postoperative delirium (POD) prediction study, detailing how heterogeneous EHR data—vitals, labs, medications, and assessments were extracted, cleaned, and structured across pre-, intra-, and postoperative stages to train machine learning models using ICDSC-based delirium labels. (PDF) [file pdig.0001267.s001.pdf]

S1 Data Description for Delirium Case Study

S1.1 Raw Data and Acquisition Stage

Patient data utilized in this study were systematically retrieved from the KISIM and PDMS at the local University Hospital between November 1, 2017, and December 31, 2022. Each patient in the dataset is assigned a unique patient ID. For different hospital visits, distinct case research IDs are allocated. A critical aspect of the dataset is the timestamp associated with each measurement, providing a chronological context to the clinical data. Meanwhile, each patient's journey throughout the hospital stay is meticulously tracked and represented through a set of unique unit codes (UnitID), distinctly identifying key locations such as the operation room, recovery room, and Intensive Care Unit (ICU).

The system categorized and coded patient statuses to accurately reflect their specific hospitalization action, encompassing categories such as:

1. PreAdmission: Denotes instances in which a patient is scheduled and awaiting bed assignment.
2. Admission: Indicating that the patient has been allocated a bed.
3. Operation: Reflecting the periods where the patient is under anesthesia.
4. Releasing: Signifying moments when the patient is ready for the transition, either to a different bed or care area.
5. Discharge: Capturing the point at which the patient leaves the ICU or the operation zone.

S1.2 Heterogeneous Data

We leverage the rich spectrum of heterogeneous and temporal electronic health record (EHR) data, encompassing pre, intra, and postoperative stages. In particular, our method integrates diverse data types including vital signs, laboratory test results, medication history, demographic details, and specific operation-related information. By harnessing these varied data sources, our model aims to capture the rich information contained in each patient's monitoring data, providing a holistic view of their risk for developing POD. Working with this complex data involves several steps and tasks and will be discussed in the following sections.

S1.2.1 Data Preprocessing

Patient and Variable Selection: We extracted patient data from the Patient Data Management System (PDMS) and the KISIM system in the local University hospital. Each patient's electronic health record is initially extracted using their unique patient ID. For each hospital visit, we utilize the distinct research case IDs assigned to the patients. This step ensures that each hospital visit is treated as a separate instance, accommodating patients with multiple visits. Then we extracted heterogeneous data from electronic health records of each hospital visit, containing demographic information, clinical nurse assessments, operation details (including type and length), and a series of intraoperative and postoperative data. This includes biometric

Table A. Statistical Analysis of Total Medication Doses Administered During Surgery and in the ICU (Excluding Non-Used Medications)

Variables	POD (n=839)	Non-POD (n=2279)	p-value
Intra-Operative Medication (total doses)			
Dexmedetomidine [mg]	0.1(0.0 – 0.1) | 42(5.0%)	0.1(0.0 – 0.2) | 156(6.8%)	0.503
Fentanyl [mcg]	500.0(300.0 – 500.0) | 651(77.6%)	500.0(300.0 – 500.0) | 1759(77.2%)	0.009
Ketamine [mg]	40.0(30.0 – 70.4) | 73(8.7%)	50.0(30.0 – 80.0) | 235(10.3%)	0.454
Midazolam [mg]	3.0(2.0 – 5.0) | 166(19.8%)	3.0(2.0 – 5.0) | 395(17.3%)	0.712
Morphine Hydrochloride Hydrate	4.0(2.0 – 5.0) | 37(4.4%)	4.0(2.0 – 6.0) | 84(3.7%)	0.461
Propofol [mg]	140.0(80.0 – 546.6) | 585(69.7%)	140.0(90.0 – 663.3) | 1601(70.3%)	0.282
Remifentanil [mg]	1220.0(556.2 – 2653.2) | 118(14.1%)	1869.0(887.5 – 2770.8) | 356(15.6%)	0.049
Post-Operative ICU Medication (total doses)			
Dexmedetomidine [mg]	0.7(0.2 – 2.0) | 336(40.0%)	0.2(0.1 – 0.6) | 328(14.4%)	< 0.001
Fentanyl [mcg]	200.0(100.0 – 350.0) | 294(35.0%)	150.0(71.2 – 275.0) | 530(23.3%)	0.718
Ketamine [mg]	130.1(50.0 – 727.2) | 48(5.7%)	658.7(99.1 – 2251.2) | 134(5.9%)	0.046
Lorazepam [mg]	2.0(1.0 – 6.0) | 125(14.9%)	1.0(1.0 – 2.5) | 123(5.4%)	< 0.001
Midazolam [mg]	5.0(2.0 – 15.1) | 205(24.4%)	15.6(5.0 – 147.1) | 286(12.5%)	< 0.001
Morphine Hydrochloride [mg]	11.0(3.0 – 20.5) | 19(2.3%)	6.0(2.0 – 11.5) | 47(2.1%)	0.628
Morphine Hydrochloride Hydrate	13.0(6.0 – 24.0) | 481(57.3%)	10.0(4.0 – 16.0) | 1142(50.1%)	< 0.001
Morphine Sulfate Pentahydrate [mg]	35.0(32.5 – 37.5) | 2(0.2%)	160.0(20.0 – 270.0) | 5(0.2%)	0.382
Oxazepam [mg]	127.5(15.0 – 146.2) | 7(0.8%)	15.0(15.0 – 24.4) | 12(0.5%)	0.011
Oxycodone [mg]	10.0(5.0 – 21.2) | 72(8.6%)	5.0(5.0 – 15.0) | 176(7.7%)	0.490
Oxycodone Hydrochloride [mg]	20.0(10.0 – 37.5) | 75(8.9%)	10.0(5.0 – 20.0) | 262(11.5%)	< 0.001

Note: Data are presented as median (interquartile range) | number of patients (%)

Table B. Statistical Analysis of Average Laboratory Testing Value During Surgery and in the ICU

Variables	POD (n=839)	Non-POD (n=2279)	p-value
Operative laboratory testing (mean)			
Basophils	0.04(0.03 – 0.06) | 254(30.3%)	0.04(0.23 – 0.07) | 482(21.1%)	0.42
Total Bilirubin	9.0(5.0 – 14.0) | 245(29.2%)	8.0(5.0 – 13.0) | 453(19.9%)	0.73
C-Reactive Protein	9.9(1.7 – 58.5) | 271(32.3%)	7.6(2.0 – 45.0) | 488(21.4%)	0.34
Eosinophils	0.04(0.01 – 0.14) | 254(30.3%)	0.04(0.01 – 0.11) | 482(21.1%)	0.69
Hemoglobin	115.0(89.8 – 128.0) | 340(40.5%)	112.0(89.0 – 130.0) | 683(30.0%)	0.97
Urea	(7.4(5.5 – 10.9) | 263(31.4%)	7.4(5.6 – 10.5) | 473(20.8%)	0.51
Creatinine	89.0(67.0 – 131.0) | 273(32.5%)	94.0(72.0 – 132.3) | 500(21.9%)	0.97
White Blood Cells	10.7(8.25 – 15.2) | 340(40.5%)	11.3(7.90 – 15.4) | 683(30.0%)	0.36
Lymphocytes	1.04(0.62 – 1.60) | 254(30.3%)	1.06(0.65 – 1.82) | 482(21.1%)	0.30
Magnesium	0.80(0.72 – 0.90) | 250(29.8%)	0.82(0.75 – 0.91) | 438(19.2%)	0.08
Monocytes	0.68(0.42 – 0.96) | 254(30.3%)	0.69(0.46 – 0.97) | 482(21.2%)	0.34
Neutrophils	9.03(6.71 – 12.8) | 254(30.3%)	9.27(6.39 – 13.5) | 482(21.2%)	0.40
Pro B-Type Natriuretic Peptide	1699(361 – 7930) | 92(11.0%)	2340(754.4 – 5098) | 155(0.07%)	0.35
Platelets	198(148 – 255.5) | 331(39.5%)	189(144.9 – 248) | 664(29.1%)	0.40
High-sensitivity Troponin T	44(18 – 92.5) | 256(30.5%)	41(21 – 102.5) | 468(20.5%)	0.60
Postoperative laboratory testing (mean)			
Basophils	0.03(0.02 – 0.05) | 818(97.5%)	0.03(0.02 – 0.05) | 2179(95.6%)	0.39
Total Bilirubin	9.0(6.0 – 16.0) | 564(67.2%)	10.5(7.0 – 18.0) | 1123(53.7%)	0.67
C-Reactive Protein	78.6(40.2 – 138.4) | 826(98.5%)	45.6(22.0 – 95.1) | 2228(97.8%)	< 0.001
Eosinophils	0.07(0.03 – 0.16) | 818(97.5%)	0.05(0.01 – 0.11) | 2179(95.6%)	< 0.001
Hemoglobin	93.5(82.2 – 110.5) | 831(99.1%)	103.5(87.7 – 118.5) | 2224(98.5%)	< 0.001
Urea	7.4(5.2 – 11.4) | 793(94.5%)	6.4(4.6 – 9.2) | 1997(87.6%)	< 0.001
Creatinine	89.0(67.0 – 131.9) | 827(98.6%)	85.0(67.5 – 116.0) | 2232(97.9%)	< 0.001
White Blood Cells	11.2(8.63 – 14.5) | 831(99.1%)	11.1(8.77 – 14.3) | 2244(98.5%)	0.95
Lymphocytes	1.02(0.72 – 1.44) | 818(97.5%)	1.02(0.72 – 1.44) | 2179(95.6%)	0.29
Magnesium	0.96(0.87 – 1.06) | 794(94.6%)	0.95(0.85 – 1.07) | 2018(88.6%)	0.46
Monocytes	0.80(0.57 – 1.07) | 818(97.5%)	0.78(0.56 – 1.03) | 2179(95.6%)	0.39
Neutrophils	8.91(6.78 – 12.0) | 818(97.5%)	9.0(6.83 – 11.9) | 2179(96.6%)	0.93
Pro B-Type Natriuretic Peptide	2563(807.9 – 6496) | 401(47.8%)	1595(538.2 – 5102.2) | 777(34.1%)	0.01
Platelets	169(123.8 – 227.2) | 831(99.1%)	170(127 – 223.4) | 2233(97.9%)	0.50
High-sensitivity Troponin T	108.5(36.1 – 603.5) | 708(84.4%)	128.5(31.0 – 557.7) | 1818(79.8%)	0.36

Note: Data are presented as median (interquartile range) | number of patients (%)

monitoring signals, laboratory test results, medication dosages, and blood gas analyses both during the operations and in the postoperative phases in recovery rooms and ICU. The detailed selected features are reported in the appendix.

Outliers Removal: We preprocessed the data by removing outliers or artifacts due to sensor errors, data entry mistakes, or physiological anomalies. The removal of these artifacts is performed using a combination of statistical methods, such as interquartile range method [1], threshold-based exclusions, and manual reviews where necessary. The goal is to ensure that the data used for analysis is as accurate and clean as possible. Variables with more than 20% missing values were excluded from consideration. Among

considered variables, any missing values were addressed using the last observation carried forward method [2,3]. In situations where a patient had no previously recorded value for a variable, we imputed the missing data with the overall average for continuous variables.

Hospital Stage Separation: We divided the patient's hospitalization journey into three stages based on their location and the actions taking place: (1) preoperation, spanning from hospital admission to the start of the operation, (2) intraoperation, the time spent in the operation, and (3) the postoperative recovery process including the ICU stay. We identify the patients who have had at least one operation during their hospital stay. The analysis is focused on the first operation and the immediate ICU stay that follows. Subsequent operations are excluded to maintain consistency in the dataset. Furthermore, a filter is applied to select patients who have an ICU stay at least one week after their first operation.

S1.2.2 Input Modalities and Study Period

Our methods incorporate multiple data modalities corresponding to different hospital stages when training our machine learning model for delirium prediction. In the preoperation stage, data consists of detailed nurse assessments, with specifics provided in the appendix. For the operation and ICU stages, the dataset includes biometric monitoring signals, laboratory test results, medication dosages, and blood gas analyses. Additionally, the ICU stage data include nurse assessments. The model utilizes data collected before the first Glasgow Coma Scale (GCS) and Intensive Care Delirium Screening Checklist (ICDSC) assessments. This approach aims to enable clinicians to predict the potential onset of delirium before these initial assessments are conducted, facilitating early detection and prevention of delirium.

S1.2.3 Task and Labelling

In this study, we utilize the Intensive Care Delirium Screening Checklist (ICDSC) [4] as the primary measure for identifying POD. The ICDSC is among the most developed and validated tools for this purpose, offering a comprehensive framework to systematically assess and diagnose delirium in a clinical setting [5]. We define a patient as being in delirium if the ICDSC score exceeds 3 at any point during a week-long ICU stay.

S1.2.4 Robust Model Building

We have designed a pipeline capable of handling the complexity of time-series data by abstracting sequential information using features' distributions. This transformation allows for the incorporation of new derived features into various machine learning models. Our pipeline is designed to facilitate the early integration of heterogeneous data sources, enhancing the model's ability to capture the dynamics of clinical contexts. To rigorously evaluate the model's performance, we implement ten-fold cross-validation ensuring a robust assessment of the model's predictive capabilities across diverse data partitions (i.e. train/test splits).

References

1. Chu X, Ilyas IF, Krishnan S, Wang J. Data cleaning: Overview and emerging challenges. In: Proceedings of the 2016 international conference on management of data; 2016. p. 2201-6.

2. Shao J, Zhong B. Last observation carry-forward and last observation analysis. Statistics in medicine. 2003;22(15):2429-41.

3. Lachin JM. Fallacies of last observation carried forward analyses. Clinical trials. 2016;13(2):161-8.

4. Krewulak KD, Rosgen BK, Ely E, Stelfox HT, Fiest KM. The CAM-ICU-7 and ICDSC as measures of delirium severity in critically ill adult patients. PLoS One. 2020;15(11):e0242378.

5. Gusmao-Flores D, Salluh JIF, Chalhub RÁ, Quarantini LC. The confusion assessment method for the intensive care unit (CAM-ICU) and intensive care delirium screening checklist (ICDSC) for the diagnosis of delirium: a systematic review and meta-analysis of clinical studies. Critical care. 2012;16:1-10.
