# Supplementary material for: Clustering of disease trajectories with explainable machine learning: A case study on postoperative delirium phenotypes
Source: PLOS Digit Health. 2026 Mar 23;5(3):e0001267. doi: 10.1371/journal.pdig.0001267 (PMC13008057; doi:10.1371/journal.pdig.0001267)
Supplement: S2 Text — details the experimental setup, including synthetic and clinical dataset protocols, a robust 10-fold cross-validation scheme repeated across 10 random seeds, AUROC/AUPRC evaluation metrics, and computational cost benchmarks for various model–SHAP explainer combinations used in the delirium prediction study. (PDF) [file pdig.0001267.s002.pdf]

S2 Experimental Settings

S2.1 Synthetic Dataset

In this experiment, we first employ a gradient boosting classifier as the prediction algorithm, trained on $\boldsymbol{X}, y$. After this classification, we compute the SHAP values across the full cohorts to uncover the feature contributions towards the predictive outcomes. To visualize the results, we leverage the t-distributed Stochastic Neighbor Embedding (t-SNE) algorithm [1]. This technique allows us to project the high-dimensional raw feature space and feature-importance space into a lower-dimensional space, facilitating the visualization of data clustering and the relationships between samples. We then apply clustering algorithms to the feature-importance space, varying the number of clusters to explore the emergence of subgroups within our synthetic dataset. This exploration aims to simulate the process of identifying latent phenotypes or subpopulations in clinical data, where the underlying subgroup structures are not directly observed. By adjusting the cluster count, we can observe the development of phenotypes.

S2.2 Peri-Operative Delirium Dataset

Based on the patient's journey through the hospital, we have divided their trajectory into three independent stages. The pre-op stage $\boldsymbol{\tau}_{\texttt{pre}}$ contains the time from hospital admission until the beginning of the first operation. The intra-op stage $\boldsymbol{\tau}_{\texttt{intra}}$ refers to the duration of the first operation itself. Lastly, the post-op stage $\boldsymbol{\tau}_{\texttt{post}}$ covers the period following the first operation up until the seventh day of the patient's stay in the Intensive Care Unit (ICU). Additionally, we introduce cumulative stages: pre^{+}, intra^{+}, and post^{+} -OP, which incorporate data from each preceding stage, respectively, allowing for a progressively comprehensive analysis of the patient's condition and risks at each step of their hospital stay. At each hospital stage, we utilize a comprehensive feature set, including static patient demographics, operation specifics, multi-scale summaries of time series distributions, and various clinical variables' measurement intensity.

To ensure the robustness and reproducibility of our results, we adopt the following experimental protocol. First, we generate ten separate train-test splits using ten different random seeds. For each split, we perform hyperparameter tuning via 10-fold cross-validation restricted solely to the training set. Finally, we repeat the entire procedure 10 times and report the average performance across these repetitions, as shown in Fig. A.

For the evaluation (i.e. testing) of our prediction models, we computed the Area Under the Receiver Operating Characteristic (AUROC) and the Area Under the Precision-Recall Curve (AUPRC) as our performance metrics. The AUROC provides a comprehensive measure of the model's ability to distinguish between classes, while the AUPRC offers valuable insights into the model's precision and recall, making them ideal for evaluating the performance of our models. The final evaluation of the trained models is based on their average performance on the test sets obtained from 10 independent train-test splits, each using a different seed.

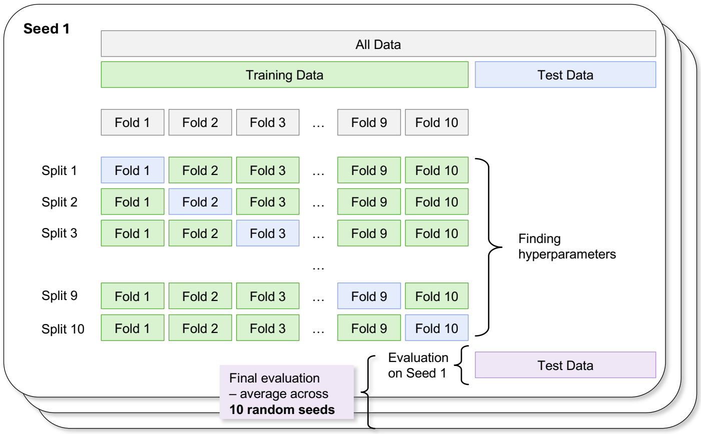

Fig A. Experimental setup for data splitting.

S2.3 Computational Cost

Table A summarizes the computational cost of model training, prediction, and SHAP explanation across different model–explainer combinations. Linear and tree-based explainers exhibit very low explanation times, highlighting their suitability for scalable clinical applications. In contrast, the KernelExplainer shows substantially higher computational cost, even when applied to a limited number of samples.

Table A. Model training, prediction times (Mean ± Std with ten repetition), and SHAP explanation times. Please note that for KernelExplainer, we only inference with 10 samples for later better visualization.

Model	Explainer Type	Training Time (s)	Prediction Time (s)	Total SHAP Computational Time per 100 Samples (s)
Logistic Regression	LinearExplainer	0.011 ± 0.011	0.000348 ± 0.000132	0.006715
MLP Classifier	KernelExplainer	1.071 ± 0.064	0.001518 ± 0.000276	7.811878 (per 10 samples)
Random Forest	TreeExplainer	0.253 ± 0.029	0.041426 ± 0.004135	1.771621
Gradient Boosting	TreeExplainer	35.193 ± 0.055	0.002745 ± 0.000879	0.009253
XGBoost	TreeExplainer	48.018 ± 5.874	0.025560 ± 0.003570	0.092225

References

1. Van der Maaten L, Hinton G. Visualizing data using t-SNE. Journal of machine learning research. 2008;9(11).
