# Supplementary material for: Clustering of disease trajectories with explainable machine learning: A case study on postoperative delirium phenotypes
Source: PLOS Digit Health. 2026 Mar 23;5(3):e0001267. doi: 10.1371/journal.pdig.0001267 (PMC13008057; doi:10.1371/journal.pdig.0001267)
Supplement: S3 Text — presents intermediate results including dimensionality reduction visualizations, cluster number selection, risk stratification across hospital stages, phenotype clustering at each perioperative stage, minimized-feature model performance, and an analysis of label noise arising from discrepancies between ICDSC and ICD-based delirium diagnoses. (PDF) [file pdig.0001267.s003.pdf]

S3 Supplementary Results and Intermediate Findings

S3.1 Clustering in the Raw Feature Space

In this section, we present the results obtained by applying various dimensionality reduction and clustering algorithms, such as t-distributed Stochastic Neighbor Embedding (t-SNE) [1], Uniform Manifold Approximation and Projection (UMAP) [2], Principal Component Analysis (PCA) [3], and Independent Component Analysis (ICA) [4] to cluster the raw features and visualize the underlying structure of the data.

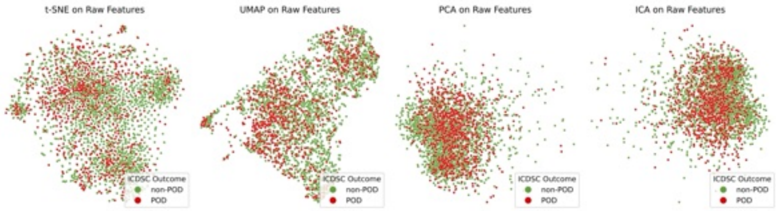

Fig A. Visualization of clustering raw features with t-SNE, UMAP, PCA, and ICA.

S3.2 Selection of number of clusters

Based on the clustering performance metrics and clinical considerations, we selected 4 clusters as the optimal number for our analysis. While 2 and 3 clusters showed slightly higher Silhouette (0.597 and 0.556) and Calinski-Harabasz scores (393.920 and 363.002), the 4-cluster solution (Silhouette: 0.556 ± 0.025, Calinski-Harabasz: 296.092 ± 48.280) provided a better balance between statistical validity and clinical interpretability. This choice was further supported by visual inspection of the clustering results and the clinical characteristics of the identified subgroups within the delirium cohort. The 4-cluster solution demonstrated stable performance across different folds, as evidenced by the relatively small standard deviations in both metrics. Moreover, increasing the number of clusters beyond 4 led to diminishing returns in terms of cluster cohesiveness and separation, with both Silhouette and Calinski-Harabasz scores showing a consistent decline from 5 clusters onward.

S3.3 Risk Stratification through Different Stages

After training our prediction models, we utilized them to calculate the individual risk probabilities (risk scores) for our patient cohort at different stages of their hospital stay. Our objective was to closely observe the evolution of patient state (that is, status) throughout the pre-operative, intra-operative, and post-operative stages, aiming to understand the progression of disease development, particularly focusing on the onset and progression of POD. We selected the best-performing model, Gradient Boosting (GB), to assess the change in the risk scores of the cohort over time. Fig. B illustrates how cohorts with different risk levels evolve across hospital stages. A major trend observed is that, prior to the onset of POD, patients predicted to be at medium or high risk are more likely to develop POD, whereas those in the low-risk cohort are less likely

Table A. Clustering Performance Metrics

Clusters	Silhouette Score (↑)	Calinski Harabasz Score (↑)
2	0.597 ± 0.049	393.920 ± 115.828
3	0.556 ± 0.022	363.002 ± 76.911
4	0.556 ± 0.025	296.092 ± 48.280
5	0.562 ± 0.020	252.394 ± 39.370
6	0.506 ± 0.110	222.545 ± 34.106
7	0.476 ± 0.130	200.164 ± 31.031
8	0.452 ± 0.140	182.811 ± 27.841
9	0.453 ± 0.138	169.095 ± 25.769
10	0.446 ± 0.144	157.761 ± 24.075
11	0.394 ± 0.172	148.319 ± 22.450
12	0.247 ± 0.143	140.170 ± 20.789

to experience POD. This indicates that our model is capable of detecting the potential for POD during the perioperative phase with a degree of accuracy. By computing these individual risk probabilities, we were able to chart the trajectory of each patient's risk level, providing a detailed view of how their likelihood of developing delirium changed from the time of hospital admission, through surgery, and into the critical post-operative period.

Fig B. Delirium risk stratification across different hospital stages.

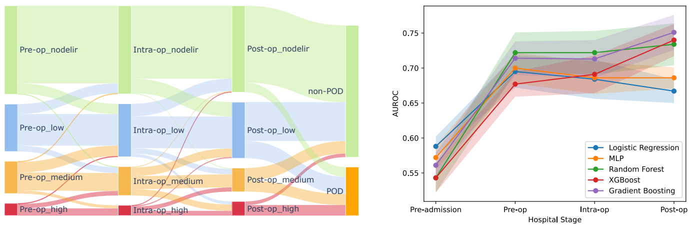

S3.4 Phenotype Clustering for Different Hospital Stages

In this section, we show intermediate results of phenotype clustering. As shown in Fig. C and D, we apply the same algorithm and pipeline to different cumulative and independent hospital stages.

S3.5 Minimized Model

In this section, we aim to refine these models to achieve a more minimalistic approach that maintains high accuracy while simplifying input heterogeneity. To accomplish this, we select the top n most important features based on their SHAP values and train our machine learning models, as shown in Table B. The primary objective is to identify a minimalistic feature set for detecting postoperative delirium, which will enable healthcare professionals to focus on fewer features and enhance the usability of the model in clinical practice. By reducing the complexity of the input data while preserving the model's performance, we strive to develop a practical and efficient tool that can be seamlessly integrated into the clinical decision-making process.

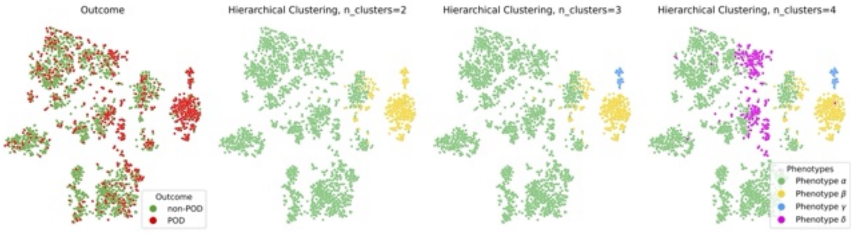

(a) Pre^{+}-OP phenotype Clustering

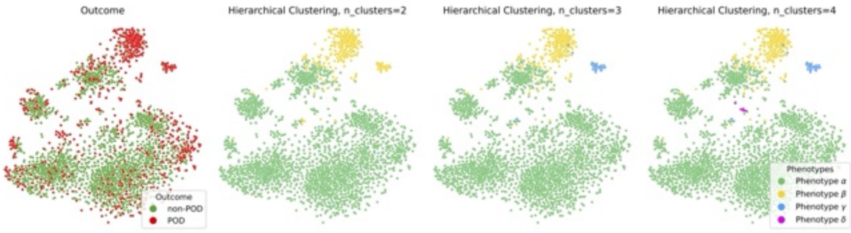

(b) Intra^{+}-OP phenotype Clustering

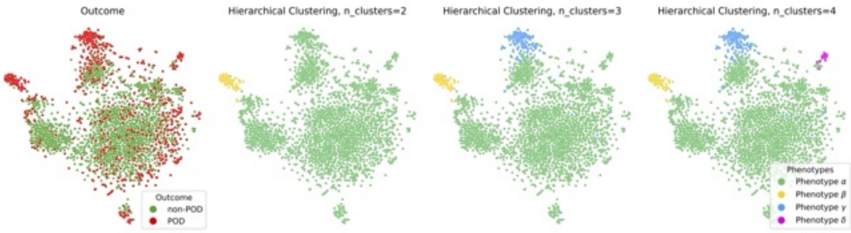

(c) Post^{+}-OP phenotype Clustering

Fig C. Phenotype Clustering for Different Hospital Stages with Different Numbers of Clusters (cumulative cases)

Table B. The auroc for different feature settings.

Stage	Model	Top 5		Top 10		Top 20		Full	
		AUROC	AUPRC	AUROC	AUPRC	AUROC	AUPRC	AUROC	AUPRC
pre^{+}-OP	Logistic Regression	0.637 ± 0.0307	0.353 ± 0.0212	0.639 ± 0.0314	0.36 ± 0.0233	0.696 ± 0.0251	0.437 ± 0.0144	0.698 ± 0.023	0.454 ± 0.022
	MLP	0.655 ± 0.0377	0.391 ± 0.0355	0.644 ± 0.036	0.366 ± 0.0322	0.694 ± 0.0288	0.434 ± 0.0263	0.704 ± 0.029	0.452 ± 0.030
	Random Forest	0.66 ± 0.0341	0.4 ± 0.038	0.669 ± 0.0351	0.409 ± 0.0401	0.715 ± 0.0289	0.497 ± 0.0357	0.721 ± 0.029	0.499 ± 0.037
	XGBoost	0.606 ± 0.0294	0.35 ± 0.0265	0.613 ± 0.0287	0.355 ± 0.0246	0.679 ± 0.0188	0.463 ± 0.0183	0.675 ± 0.014	0.445 ± 0.019
	Gradient Boost	0.657 ± 0.0324	0.38 ± 0.0253	0.663 ± 0.0353	0.392 ± 0.0328	0.713 ± 0.0239	0.484 ± 0.0226	0.714 ± 0.026	0.485 ± 0.029
intra^{+}-OP	Logistic Regression	0.661 ± 0.028	0.366 ± 0.0169	0.685 ± 0.0261	0.413 ± 0.0188	0.699 ± 0.0246	0.449 ± 0.0175	0.666 ± 0.029	0.427 ± 0.035
	MLP	0.664 ± 0.0319	0.383 ± 0.0311	0.691 ± 0.0304	0.43 ± 0.0271	0.697 ± 0.0231	0.458 ± 0.0195	0.669 ± 0.030	0.429 ± 0.029
	Random Forest	0.677 ± 0.0305	0.433 ± 0.0247	0.713 ± 0.0278	0.498 ± 0.0364	0.725 ± 0.0284	0.503 ± 0.0307	0.712 ± 0.034	0.474 ± 0.036
	XGBoost	0.631 ± 0.0199	0.383 ± 0.0171	0.683 ± 0.0238	0.456 ± 0.0154	0.688 ± 0.0203	0.461 ± 0.0267	0.690 ± 0.024	0.454 ± 0.033
	Gradient Boost	0.687 ± 0.0278	0.435 ± 0.0315	0.719 ± 0.024	0.487 ± 0.0228	0.721 ± 0.0251	0.49 ± 0.0257	0.704 ± 0.027	0.466 ± 0.029
post^{+}-OP	Logistic Regression	0.657 ± 0.0261	0.362 ± 0.0157	0.684 ± 0.0246	0.415 ± 0.0179	0.702 ± 0.024	0.472 ± 0.0192	0.659 ± 0.023	0.428 ± 0.033
	Multilayer Perceptron	0.676 ± 0.042	0.405 ± 0.0549	0.69 ± 0.0329	0.428 ± 0.032	0.707 ± 0.0246	0.48 ± 0.0266	0.667 ± 0.025	0.433 ± 0.035
	Random Forest	0.681 ± 0.0333	0.447 ± 0.0233	0.713 ± 0.0266	0.498 ± 0.033	0.752 ± 0.0229	0.534 ± 0.0317	0.724 ± 0.031	0.490 ± 0.037
	XGBoost	0.634 ± 0.0235	0.388 ± 0.0209	0.678 ± 0.0157	0.452 ± 0.0167	0.732 ± 0.0159	0.521 ± 0.0209	0.731 ± 0.021	0.512 ± 0.028
	Gradient Boosting	0.689 ± 0.0335	0.445 ± 0.0301	0.720 ± 0.0228	0.494 ± 0.0308	0.761 ± 0.0203	0.551 ± 0.0248	0.743 ± 0.019	0.533 ± 0.029

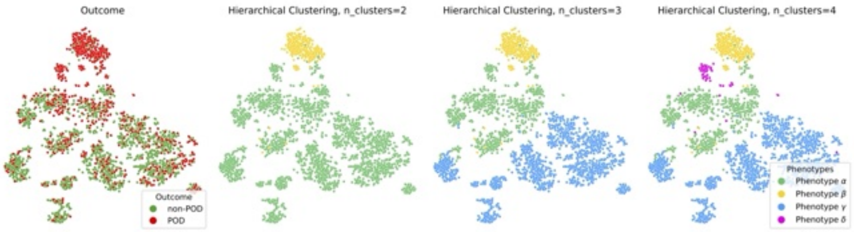

(a) Pre-OP phenotype Clustering

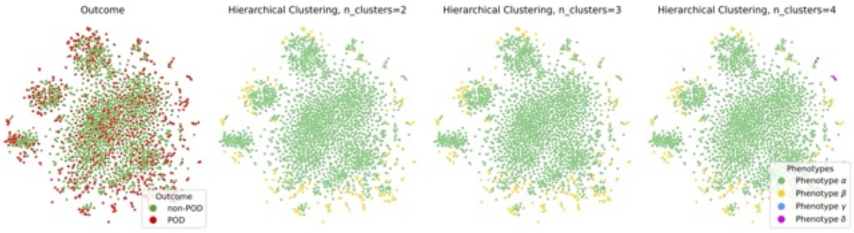

(b) Intra-OP phenotype Clustering

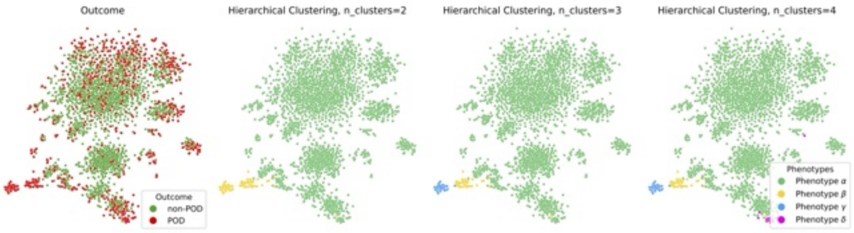

(c) Post-OP phenotype Clustering

Fig D. Phenotype Clustering for Different Hospital Stages with Different Numbers of Clusters (independent cases)

S3.6 Different cases regarding ICU delirium

In this section, we delve into patient records to categorize them into distinct cohorts, aiming to assess the reliability of our delirium labeling process. Utilizing both the Intensive Care Delirium Screening Checklist (ICDSC) during ICU stays and the International Classification of Diseases (ICD) codes assigned post-hospital discharge, we aim to dissect the intricacies of delirium diagnosis. The ICDSC is employed by healthcare professionals in the ICU to screen for delirium symptoms systematically, while ICD codes offer a global standard for recording diagnoses and health conditions post-discharge.

Patients are categorized into four groups based on the presence or absence of these indicators:

- **Confirmed ICU Delirium**: Patients with positive indicators from both ICDSC and ICD codes are classified as having confirmed ICU delirium.
- **ICU Delirious Symptoms**: Patients showing positive ICDSC results but without corresponding positive ICD codes are considered to exhibit delirium symptoms specifically in the ICU, suggesting potential underdiagnosis post-discharge.
- **Ward Delirium**: Patients with negative ICDSC but positive ICD codes are identified as experiencing delirium recognized outside the ICU, possibly indicating delirium that developed or was diagnosed later in the ward.
- **No Delirium**: Patients lacking positive results from both ICDSC and ICD codes are categorized as not having experienced delirium.

As shown in Fig E, different assessment procedures of labeling delirium highlights the intricate and possibly imprecise nature of these labels, which mirrors the challenges faced by healthcare professionals in accurately diagnosing and documenting delirium throughout various stages of patient care. The findings from this analysis emphasize the need for addressing the issue of **exsiting noise in delirium labels** when training a machine learning model. Moreover, these insights underscore the significance of refining and improving our current methods for detecting and categorizing delirium to ensure more precise and reliable diagnoses in the future.

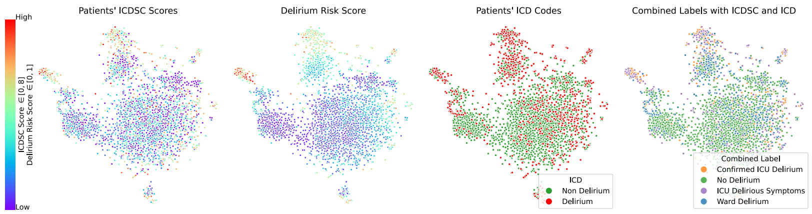

Fig E. Development of subgroups through different hospital stages

References

1. Van der Maaten L, Hinton G. Visualizing data using t-SNE. Journal of machine learning research. 2008;9(11).
2. McInnes L, Healy J, Melville J. Umap: Uniform manifold approximation and projection for dimension reduction. arXiv preprint arXiv:180203426. 2018.

3. Wold S, Esbensen K, Geladi P. Principal component analysis. Chemometrics and intelligent laboratory systems. 1987;2(1-3):37-52.

4. Hyvärinen A, Oja E. Independent component analysis: algorithms and applications. Neural networks. 2000;13(4-5):411-30.
